# Increased Circulating Levels of Ectodysplasin A in Newly Diagnosed Type 2 Diabetic Patients

**DOI:** 10.3389/fendo.2021.737624

**Published:** 2021-11-09

**Authors:** Xia Deng, Zhensheng Cai, Yanyan Li, Xunan Wu, Li Zhao, Haoxiang Li, Ke Chen, Panpan Zhang, Chenxi Wang, Zhicong Zhao, Ling Yang, Guoyue Yuan

**Affiliations:** ^1^ Department of Endocrinology, Affiliated Hospital of Jiangsu University, Zhenjiang, China; ^2^ Department of Endocrinology, Shanghai Pudong Hospital, Fudan University, Shanghai, China

**Keywords:** ectodysplasin A, T2DM, insulin resistance, hepatokine, hyperlipidemia

## Abstract

**Objective:**

Ectodysplasin A (EDA), a newly discovered hepatokine, has recently been considered to be closely related to glycolipid metabolism disorders, but the pathophysiological effects of EDA are still poorly understood. This study was the first time to determine the level of serum EDA in newly diagnosed type 2 diabetes mellitus (T2DM) patients, and to explore the relationships between serum EDA levels and various metabolic indexes.

**Methods:**

A total of 184 subjects were enrolled in the study, including 92 subjects with newly diagnosed T2DM and 92 subjects with age- and sex-matched normal glucose tolerance (NGT). Serum EDA levels were determined using enzyme-linked immunosorbent assay (ELISA). Oral glucose tolerance test, glycosylated hemoglobin c (HbA1c), and insulin were also measured.

**Results:**

Serum EDA levels were significantly increased in the T2DM group than in the NGT group (359.91 ± 117.99 vs. 265.82 ± 86.51 pg/ml, *p* < 0.001). Serum EDA levels were positively correlated with body mass index (BMI), waist-to-hip ratio (WHR), fasting plasma glucose (FPG), HbA1c, 2-hour postprandial plasma glucose (2hPG), fasting plasma insulin (FIns), fasting C peptide (FCP), triglyceride (TG), HOMA-IR, and negatively correlated with high-density lipoprotein cholesterol (HDL-c) and HOMA-β (*p* < 0.05). Multiple stepwise regression analysis demonstrated that 2hPG and FIns were independent influencing factors of serum EDA level (*p* < 0.05). Logistic regression analysis showed that serum EDA level was significantly independently correlated with T2DM (*p* < 0.05).

**Conclusions:**

Serum EDA levels are significantly higher in T2DM patients, suggesting that EDA may play a role in the occurrence and development of T2DM.

## Introduction

Diabetes is a group of metabolic diseases characterized by chronic hyperglycemia caused by a variety of reasons, of which 90% are type 2 diabetes mellitus (T2DM), and the main pathophysiological mechanisms are insulin resistance and relatively insufficient insulin secretion (1, 2). Liver is the central organ of glucose and lipid metabolism, which plays an important role in the development of T2DM (3). Hepatic insulin resistance is considered to be the main driving factor of insulin resistance (4). Previous studies have shown that the most obvious pathophysiological characteristics of hepatic insulin resistance were gluconeogenesis and glycolysis dysfunction, and liver lipid accumulation (5). However, in addition to the role of glucose and lipid metabolism, the latest research also showed that the liver is an important endocrine organ, which can secrete thousands of proteins, of which about 25% can be released into the blood circulation (6). Further studies have found that a variety of protein factors secreted by the liver form a regulatory network and affect the energy metabolism of the liver and other organs through inter tissue communication (7), thus affecting the occurrence and development of metabolic-related diseases, such as obesity, insulin resistance, diabetes, and fatty liver (8–11). A quantitative protein expression profile based on isobaric tagging for relative and absolute quantification (iTRAQ) showed that there were 69 differentially expressed proteins in the plasma of T2DM patients compared with non-diabetic individuals, including a variety of proteins secreted by the liver and related to insulin resistance in diabetic patients, including α 2-macroglobulin, selenoprotein P, retinol binding protein 4 (RBP4) (12).

Ectodysplasin A (EDA), a newly discovered hepatokine, is closely related to chronic diseases such as fatty liver, obesity, and insulin resistance (13, 14). It is mainly secreted by hepatocytes *in vivo*, and is significantly higher than that in white adipose tissue, brown adipose tissue, skeletal muscle cells, and other tissues (14). The gene was initially considered as a member of tumor necrosis factor (TNF)-related cytokine family, belonging to type II transmembrane protein, which can be secreted into the extracellular domain after cleavage of endoprotease furan (15). Previous studies have shown that EDA plays an important role in the development and maintenance of skin-derived structures such as teeth, hair, and sweat glands, and EDA gene mutations can lead to X-linked hypohidrotic ectodermal dysplasia (16) and selective nonsyndromic tooth dysplasia (17). With the deepening of research, in 2017, Awazawa et al. claimed to have found a new function of this gene expression—regulated systemic glucose metabolism, and led to impaired insulin sensitivity of skeletal muscle, which was considered as a hepatokine. In that study, the results showed that the liver and serum levels of EDA were significantly increased in high-fat diet (HFD) mice and db/db mice. What is more, it was found that overexpression of EDA exacerbated the impaired glucose tolerance in mice, while knockdown of EDA significantly improved insulin sensitivity in db/db mice (14).

Although knockdown EDA has strong anti-diabetic properties, the exact understanding of its biological activity and mode of action remains to be further studied. In order to explore the clinical significance of EDA in human, we measured the serum concentration of EDA in healthy control subjects and newly diagnosed T2DM patients and analyzed the relationship between EDA concentration and anthropometric and metabolic parameters.

## Research Design and Methods

### Study Population

A total of 184 adults were recruited: 92 subjects with normal glucose tolerance (NGT) and 92 patients with T2DM matched with gender and age. None of the healthy controls took drugs known to affect glucose tolerance and lipid metabolism. All patients with T2DM were newly diagnosed and did not take oral hypoglycemic and hypolipidemic drugs. The diagnosis of T2DM was based on the diagnostic criteria of the American Diabetes Association in 2011 (18). Patients with impaired fasting glucose and/or impaired glucose tolerance, type 1 diabetes mellitus, gestational diabetes mellitus, active hepatitis/cirrhosis, acute liver failure, hemodialysis chronic renal failure, congestive heart failure, or other known major diseases were excluded from the study. Each subject was asked about smoking and alcohol consumption. Approval for the study was obtained from the Clinical Research Ethics Committee, Affiliated Hospital of Jiangsu University. Informed consent was obtained from each of the participants.

### Anthropometric and Biochemical Measurements

General anthropometric parameters such as height, weight, waist circumference, hip circumference, and blood pressure were collected and recorded by professional doctors, and BMI [weight (kg)/the square of height (m)] and WHR (the ratio of waist circumference to hip circumference) were calculated. The plasma glucose at each time point of OGTT was measured by glucose oxidase-based assay, and the level of insulin and c-peptide was detected by chemiluminescence method. The high-performance liquid chromatography (HPLC) (Arkray Inc., Kyoto, Japan) method was used to detect the glycosylated hemoglobin (HbA1c). The high-density lipoprotein cholesterol (HDL-c), low-density lipoprotein cholesterol (LDL-c), total cholesterol (TC), and triglyceride (TG) parameters of blood lipid profile were measured by appropriate enzymatic assays (Beckman Coulter Inc., Brea, CA, USA). The hepatic insulin resistance was estimated by the homeostasis model assessment (HOMA): HOMA-IR = Fins × FPG/22.5 (19). The β-cell function was estimated by the HOMA of β-cell function: HOMA-β = 20 × FIns/(FPG-3.5).

### Estimation of Serum EDA Levels

The fasting blood samples of each subject were collected and centrifuged immediately at 1,000×*g* at 4°C for 20 min, and then the serum samples were separated and labeled, and stored in a −80°C refrigerator. Serum EDA levels were determined using a commercially available human enzyme-linked immunosorbent assay (ELISA) (Wuhan Eiaab Science Co., China; Catalog No. E1976h). The sensitivity of the kit was less than 20 pg/ml, the intra-assay CV was ≤7.8%, and the inter-assay CV was ≤8.9%. The operation process was carried out according to the instructions of the kit. The absorbance value at 450 nm was detected by enzyme-labeled instrument (ThermoFisher, Multiskan GO), and the standard curve was drawn. Furthermore, the concentration of the sample was calculated by using the standard curve. The detection range of ELISA was 78–5,000 pg/ml.

### Diagnosis of Non-Alcoholic Fatty Liver Disease

Abdominal ultrasound (full-body color Doppler diagnostic instrument LOGIQ-9) was performed by a professional ultrasound doctor. The diagnosis of NAFLD was based on the “Guidelines for the Diagnosis and Treatment of Nonalcoholic Fatty Liver Diseases” in 2012 (20).

### Statistical Analysis

All statistical analyses were performed using SPSS version 20.0. Continuous variables were expressed as mean ± SD and median (quartile) or categorical variables as cases (percentage). ALT, AST, GGT, HOMA-IR, and HOMA-β values were converted logarithmically due to their nonnormal distribution. The differences between the two groups were compared by independent Student’s *t* test. The categorical variables were tested by *χ* ^2^ test. One-way ANOVA test was used for multiple comparisons. Pearson correlation analysis was used to evaluate the associations between serum EDA and various variables. After adjusting for the effects of gender, age, and BMI, the correlation was analyzed by partial correlation. The independent influencing factors of EDA were analyzed by linear stepwise regression. Binary logistic regression analysis was used to examine the significant trend of the increase in the tertiles, and the lowest tertile was used as a reference category to estimate the odds ratio of diabetes in each tertile. A double-tailed test value of *p* < 0.05 was considered statistically significant.

## Results

### The Clinical and Biochemical Parameters in NGT and T2DM Groups

**Table 1** summarizes the clinical baseline characteristics of the study subgroups. There was no significant difference in gender, age, smoking history, alcohol consumption, and TC between the T2DM group and NGT group. In addition, compared with the NGT group, BMI, WHR, SBP, DBP, HbA1c, FPG, 2hPG, FIns, FCP, TG, LDL-c, ALT, AST, GGT, and HOMA-IR in the T2DM group were significantly increased (*p* < 0.05), while HDL-c and HOMA-β were significantly reduced (*p* < 0.05).

**Table 1:** Clinical and biochemical characteristics in control subjects and in patients with T2DM.

Parameters	NGT (*n* = 92)	T2DM (*n* = 92)	*p-*value
Age (years)	47.016 ± 11.61	48.30 ± 11.07	0.440
Sex, female (%)	53 (57.6%)	50 (54.3%)	0.656
BMI (kg/m^2^)	23.89 ± 3.66	25.89 ± 3.43	<0.001
WHR	0.88 ± 0.06	0.93 ± 0.05	<0.001
SBP (mmHg)	120.03 ± 15.50	129.90 ± 14.31	<0.001
DBP (mmHg)	72.91 ± 13.31	82.50 ± 10.66	<0.001
Smoking status, *n* (%) Never Former smoker Current smoker	66 (71.7%) 5 (5.4%) 21 (22.8%)	62 (67.4%) 7 (7.6%) 23 (25.0%)	0.522 0.550 0.730
Alcohol use, *n* (%) Never Occasional drinker Regular drinker	77 (83.7%) 6 (6.5%) 9 (9.8%)	71 (77.2%) 10 (10.9%) 11 (12.0%)	0.265 0.295 0.636
HbA1c (%)	5.71 ± 0.29	9.78 ± 1.88	<0.001
FPG (mmol/L)	4.87 ± 0.55	11.09 ± 2.81	<0.001
2hPG (mmol/L)	6.23 ± 0.92	20.22 ± 4.30	<0.001
FIns (µIU/ml)	5.69 ± 4.11	8.21 ± 4.62	<0.001
FCP (ng/ml)	2.29 ± 0.88	3.09 ± 0.93	<0.001
TG (mmol/L)	1.56 ± 0.85	2.54 ± 1.44	<0.001
TC (mmol/L)	4.97 ± 0.85	5.12 ± 1.06	0.280
LDL-c (mmol/L)	2.81 ± 0.75	3.08 ± 0.87	0.026
HDL-c (mmol/L)	1.44 ± 0.41	1.07 ± 0.29	<0.001
ALT (U/L)	16.15 (10.63–31.38)	33.3 (18.13–50.05)	<0.001
AST (U/L)	17.15 (14.25–20.88)	20.3 (15.28–31.23)	0.003
GGT (U/L)	20.8 (15–30.75)	37 (25–60.5)	<0.001
HOMA-IR	1.02 (0.65–1.48)	3.47 (2.37–4.85)	<0.001
HOMA-β	70.10 (45.46–123.11)	18.45 (11.12–34.39)	<0.001
Ectodysplasin A (pg/ml)	265.82 ± 86.51	359.91 ± 117.99	<0.001

Data are presented as means ± SD, medians [interquartile range (IQR)], and number (percentages).

BMI, body mass index; WHR, waist-to-hip ratio; SBP, systolic blood pressure; DBP, diastolic blood pressure; HbA1c, glycosylated hemoglobin c; FPG, fasting plasma glucose; 2hPG, 2-hour postprandial plasma glucose; FIns, fasting plasma insulin; FCP, fasting C peptide; TG, triglyceride; TC, total cholesterol; LDL-c, low-density lipoprotein cholesterol; HDL-c, high-density lipoprotein cholesterol; ALT, alanine aminotransferase; AST, aspartate aminotransferase; GGT, g-glutamyl transpeptidase; HOMA-IR, homeostasis model assessment-insulin resistance index; HOMA-β, homeostasis model assessment-β.

**Figure 1:**
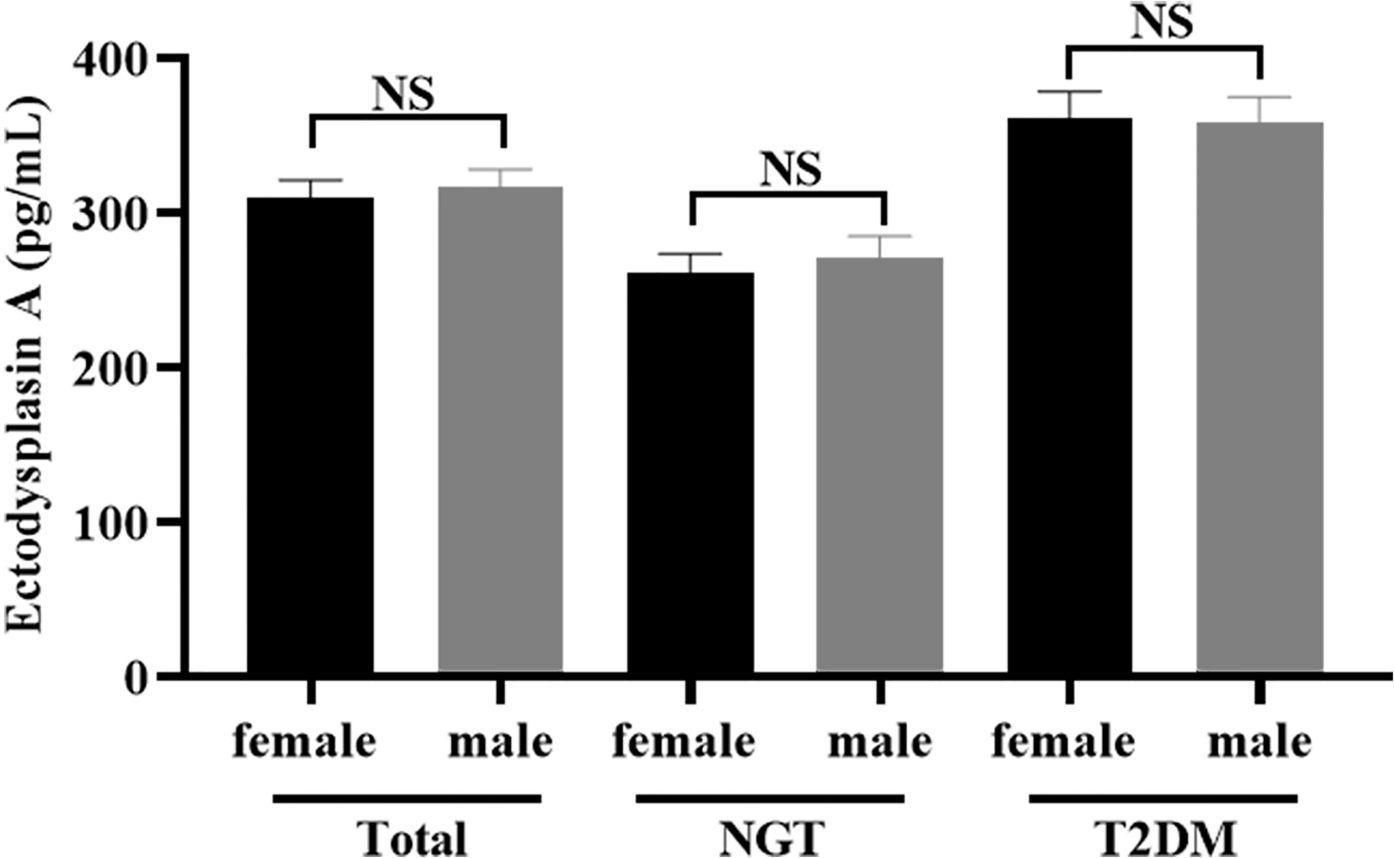
Comparison of the serum concentration of EDA between women and men. No significant (NS): P > 0.05 was considered as no significant difference.

Most importantly, the serum level of EDA in the T2DM group was significantly increased than that in the NGT group (265.82 ± 86.51 vs. 359.91 ± 117.99 pg/ml), and the difference was statistically significant (*p* < 0.001) (**Table 1**). However, no difference in serum EDA levels was observed between men and women in T2DM (360.80 ± 128.35 vs. 358.85 ± 105.87 pg/ml, *p* = 0.938) or NGT (261.62 ± 87.85 vs. 271.53 ± 85.46 pg/ml, *p* = 0.590) groups (**Figure 1**).

### Estimated Non-Alcoholic Fatty Liver Disease in Study Subjects

In the present study, 23 subjects had missing data on abdominal ultrasound for NAFLD. For the remaining 161 patients, BMI, WHR, SBP, DBP, HbA1c, FPG, 2hPG, FIns, FCP, TG, TC, LDL-C, HDL-C, ALT, AST, GGT, and HOMA-IR in patients with NAFLD were significantly higher than the non-NAFLD group (*p* < 0.05); however, HDL-c and HOMA-β were significantly lower than the control group (*p* < 0.05). Interestingly, the serum level of EDA in the NAFLD group was significantly increased than that in the non-NAFLD group (269.24 ± 88.08 vs. 345.07 ± 106.80 pg/ml), and the difference was statistically significant (*p* < 0.001) (**Table 2**).

**Table 2:** Clinical and biochemical characteristics in NAFLD and non-NAFLD groups.

Parameters	Non-NAFLD (*n* = 81)	NAFLD (*n* = 80)	*p-*value
Age (years)	48.54 ± 11.36	47.75 ± 11.28	0.440
Sex, female (%)	31 (38.3%)	40 (50.0%)	0.134
BMI (kg/m^2^)	22.97 ± 3.06	26.41 ± 3.39	<0.001
WHR	0.87 ± 0.06	0.93 ± 0.05	<0.001
SBP (mmHg)	119.37 ± 15.34	130.05 ± 15.06	<0.001
DBP (mmHg)	72.91 ± 13.17	81.38 ± 11.48	<0.001
Smoking status, *n* (%) Never Former smoker Current smoker	60 (74.1%) 18 (22.2%) 3 (3.7%)	53 (66.3%) 20 (25.0%) 7 (8.8%)	0.817 0.678 0.185
Alcohol use, *n* (%) Never Occasional drinker Regular drinker	69 (85.2%) 7 (8.6%) 5 (6.2%)	63 (78.8%) 8 (10.0%) 9 (11.3%)	0.212 0.767 0.253
HbA1c (%)	6.25 ± 1.64	9.21 ± 2.30	<0.001
FPG (mmol/L)	5.54 ± 2.26	10.36 ± 3.42	<0.001
2hPG (mmol/L)	8.08 ± 5.40	17.99 ± 6.73	<0.001
FIns (µIU/ml)	4.92 ± 2.76	8.78 ± 5.10	<0.001
FCP (ng/ml)	2.15 ± 0.80	3.18 ± 0.93	<0.001
TG (mmol/L)	1.50 ± 0.83	2.49 ± 1.36	<0.001
TC (mmol/L)	4.91 ± 0.85	5.23 ± 0.99	0.031
LDL-c (mmol/L)	2.76 ± 0.72	3.16 ± 0.85	0.002
HDL-c (mmol/L)	1.44 ± 0.44	1.11 ± 0.30	<0.001
ALT (U/L)	16.00 (10.15–30.40)	33.30 (16.78–52.20)	0.001
AST (U/L)	17.10 (13.80–20.75)	19.80 (15.78–30.38)	0.029
GGT (U/L)	19.00 (14.00–28.45)	37.00 (25.00–61.00)	<0.001
HOMA-IR	0.99 (0.64–1.47)	3.50 (2.13–4.85)	<0.001
HOMA-β	58.78 (39.11–97.99)	23.59 (13.13–44.11)	0.001
Ectodysplasin A (pg/ml)	269.24 ± 88.08	345.07 ± 106.80	<0.001

### Clinical Features of the Participants According to the Tertiles of EDA

Compared with the lower serum EDA tertile group, prevalence of diabetes, BMI, WHR, HbA1c, FPG, 2hPG, FIns, FCP, TG, and HOMA-IR in the middle or upper serum EDA tertile group were significantly increased (*p* < 0.05), while HDL-c was significantly decreased (*p* < 0.05). Other metabolic parameters, such as sex, age, smoking history, alcohol consumption, SBP, DBP, TC, LDL-c, ALT, AST, GGT, and HOMA-β did not reach statistical significance (**Table 3**).

**Table 3:** Clinical and biochemical characteristics of the study subjects according to the tertiles of Ectodysplasin A.

Parameters	Lower Tertile (*n* = 62)	Middle Tertile (*n* = 62)	Upper Tertile (*n* = 60)	*p-*value
Age (years)	48.35 ± 11.71	47.89 ± 10.41	46.70 ± 11.95	0.711
Sex, female(%)	38 (61.3%)	32 (51.6%)	33 (55.0%)	0.545
BMI (kg/m^2^)	24.42 ± 3.43	24.39 ± 3.42	25.91 ± 4.01^ab^	0.033
WHR	0.89 ± 0.06	0.90 ± 0.07	0.92 ± 0.06^b^	0.041
SBP (mmHg)	123.95 ± 15.70	125.35 ± 17.22	125.62 ± 14.12	0.820
DBP (mmHg)	76.15 ± 13.81	78.34 ± 13.04	78.67 ± 11.98	0.504
Smoking status, *n* (%) Never Former smoker Current smoker	47 (75.8%) 3 (4.8%) 12 (19.4%)	42 (67.7%) 3 (4.8%) 17 (27.4%)	39 (65.0%) 6 (10.0%) 15 (25.0%)	0.401 0.449 0.558
Alcohol use, *n* (%) Never Occasional drinker Regular drinker	51 (82.3%) 4 (6.5%) 7 (11.3%)	50 (80.6%) 5 (8.1%) 7 (11.3%)	47 (78.3%) 7 (11.7%) 6 (10.0%)	0.860 0.579 0.966
HbA1c (%)	7.02 ± 2.31	7.68 ± 2.39	8.56 ± 2.41^ab^	0.002
FPG (mmol/L)	6.84 ± 3.60	8.02 ± 3.77	9.11 ± 3.49^b^	0.003
2hPG (mmol/L)	10.73 ± 7.40	13.13 ± 7.75	15.90 ± 7.06^ab^	0.001
FIns (µIU/ml)	5.19 ± 2.65	7.01 ± 4.30^a^	8.70 ± 5.59^ab^	<0.001
FCP (ng/ml)	2.26 ± 0.80	2.79 ± 1.07^a^	3.04 ± 0.91^a^	<0.001
TG (mmol/L)	1.72 ± 1.27	2.06 ± 1.12	2.37 ± 1.36^a^	0.019
TC (mmol/L)	4.97 ± 1.00	5.08 ± 0.81	5.09 ± 1.07	0.733
LDL-c (mmol/L)	2.86 ± 0.91	2.99 ± 0.70	2.99 ± 0.84	0.582
HDL-c (mmol/L)	1.36 ± 0.38	1.26 ± 0.45	1.14 ± 0.34^a^	0.008
ALT (U/L)	18.6 (12.9–37.88)	22.65 (14.15–38.48)	28.65 (15.2–47.8)	0.461
AST (U/L)	17.7 (13.95–24.23)	17.2 (14.5–23)	19.9 (15.95–29.35)	0.230
GGT (U/L)	25 (15–41.5)	28.5(19–53.25)	29 (21.25–50)	0.164
HOMA-IR	1.27 (0.78–2.17)	1.81 (1.00–3.62)^a^	3.27 (1.80–4.70)^ab^	<0.001
HOMA-β	47.84 (19.72–105.31)	41.10 (22.77–77.68)	33.84 (15.88–53.41)	0.154
Ectodysplasin A (pg/ml)	200.53 ± 40.28	301.73 ± 31.00^a^	440.46 ± 85.43^ab^	<0.001
T2DM, *n* (%)	18 (29.0%)	31 (50.0%)^a^	43 (71.7%)^ab^	<0.001

a Significant p ≤0.05 vs. group of lower tertile.

b Significant p ≤ 0.05 vs. group of middle tertile.

### Correlation of EDA With Metabolic Parameters

Serum EDA concentrations were positively correlated with BMI, WHR, HbA1c, FPG, 2hPG, FIns, FCP, TG, and HOMA-IR, but inversely correlated with HOMA-β and HDL-c. In addition, after adjusting for gender, age, and BMI, the HbA1c, FPG, 2hPG, FIns, TG, HDL-c, HOMA-IR, and HOMA-β were still significantly correlated with serum EDA levels (**Table 4**). Furthermore, stepwise multiple linear regression models revealed that 2hPG and FIns were independently related to the serum EDA levels (**Table 5**).

**Table 4:** Partial correlations analysis of variables associated with serum Ectodysplasin A levels in study population.

Parameters	EDA (unadjusted)		EDA (age, BMI, and sex adjusted)	
	*r*	*p*	*r*	*p*
Age	−0.083	0.261	–	–
BMI	0.235	0.001	–	–
WHR	0.150	0.042	0.037	0.628
SBP	0.070	0.348	−0.025	0.743
DBP	0.118	0.110	0.027	0.716
HbA1c	0.321	<0.001	0.295	<0.001
FPG	0.325	<0.001	0.256	0.001
2hPG	0.363	<0.001	0.336	<0.001
FIns	0.284	<0.001	0.192	0.010
FCP	0.277	<0.001	0.184	0.014
TG	0.231	0.002	0.172	0.022
TC	0.111	0.134	0.122	0.104
LDL-c	0.116	0.118	0.103	0.170
HDL-c	−0.259	<0.001	−0.174	0.020
ALT	0.101	0.173	0.037	0.622
AST	0.102	0.169	0.065	0.386
GGT	0.128	0.082	0.065	0.388
HOMA-IR	0.352	<0.001	0.277	<0.001
HOMA-β	−0.184	0.012	−0.231	0.002

**Table 5:** Stepwise multiple linear regression analysis with Ectodysplasin A as the dependent variable.

Independent Variable	Regression Coefficient (SE)	*β*	95% CI	*p*
(Constant)	209.528 (17.97)	–	174.075–244.982	<0.001
2hPG	4.796 (1.01)	0.32	2.806–6.785	<0.001
FIns	5.745 (1.70)	0.23	2.385–9.106	0.001

CI, confidence interval.

The following independent variables were considered for the model: age, BMI, WHR, HbA1c, FPG, 2hPG, FIns, FCP, TC, TG, LDL-c, HDL-c, HOMA-IR, HOMA-β, ALT, AST, and GGT. Only the variables that had a p < 0.05 were considered in the final fitted model.

### Logistic Regression Analyses for T2DM

The prevalence of T2DM was 71.7%, 50.0%, and 29.0% in the high, medium, and low EDA concentration groups, respectively (*χ* ^2^ = 22.170, *p* < 0.001) (**Table 3**). Binary logistic regression analyses demonstrated that EDA was significantly associated with T2DM (*p* < 0.05 in unadjusted model) (**Table 6**, model 1). Moreover, EDA was significantly associated with T2DM even after adjustment for age, sex, BMI, WHR, SBP, DBP, and liver function (*p* < 0.05) (**Table 6**, model 2).

**Table 6:** OR and 95% CI for T2DM by the tertiles of Ectodysplasin A.

Models	EDA (pg/ml)	Individuals With and Without T2DM			
		*n*	OR	95% CI	*p*
Model 1	Lower Tertile	62	1	Ref	Ref
	Middle Tertile	62	2.783	1.166–5.127	0.018
	Upper Tertile	60	6.183	2.821–13.554	<0.001
Model 2	Lower Tertile	62	1	Ref	Ref
	Middle Tertile	62	2.783	1.118–6.927	0.028
	Upper Tertile	60	7.216	2.758–18.885	<0.001

CI, confidence interval; OR, odds ratio.

Adjusted for age, sex, BMI, WHR, SBP, DBP, ALT, and AST.

## Discussion

To our knowledge, this is the first time to investigate the relationship between circulating EDA levels and T2DM. Our results showed that serum EDA levels were significantly higher in newly diagnosed and untreated T2DM patients compared with healthy controls. Serum EDA concentration was positively correlated with BMI, WHR, HbA1c, FPG, 2hPG, fins, FCP, TG, and HOMA-IR, but negatively correlated with HOMA-β and HDL-C even after age and sex adjustment. What is more, the prevalence of T2DM was more obvious in the upper serum EDA tertile group. Nevertheless, our findings did not accord with the recent study performed by Bayliss et al. (21) in which serum EDA was not elevated in patients with T2DM and had nothing to do with FPG, HbA1c, and HOMA-IR in obese individuals. The differences may be caused by different sample sizes, populations, reagents, and disease duration of T2DM. In that study, patients with NAFLD and normal subjects were recruited from individuals undergoing laparoscopic adjustable gastric band, sleeve gastrectomy, or gastric bypass surgery, and all patients had BMI ≥ 30 kg/m^2^ and abnormal liver function. Obesity and abnormal liver function may interfere with the relationship between EDA and insulin resistance as well as glucose metabolism. In addition, there was not enough detailed information about T2DM. Whether oral hypoglycemic and hypolipidemic drugs will affect serum EDA level is unclear. In our study, subjects treated with oral hypoglycemic or hypolipidemic drugs were excluded to avoid possible confounding effects of drugs, which increased the credibility. Of course, there were similarities between the two studies. They both found that EDA levels were higher in patients with NAFLD than that in non-NAFLD patients.

The mechanisms underlying increased EDA levels in newly diagnosed T2DM patients remain unclear. Hepatokines are secreted by hepatocytes that can influence multiple metabolic processes through autocrine, paracrine, and endocrine signaling (22). Previous studies have found that a variety of hepatokines play an important role in the occurrence and development of insulin resistance and T2DM, including fibroblast growth factor 21 (FGF21), retinol binding protein 4 (RBP4), and fetuin A (7, 23, 24). In our study, the serum level of EDA, a newly discovered hepatokine, in subjects with the T2DM was much higher than those without this disorder, and serum EDA level was significantly independently correlated with T2DM. Most importantly, 2hPG and FIns were independently interfering factors of EDA. This result suggests that elevated serum EDA can be potentially used as a risk factor for T2DM. Of course, further research is necessary to clarify its possible mechanism. Recently, Awazawa et al. (14)reported that hepatic expression of EDA was upregulated in animal models of diabetes and obesity. Further studies showed that the overexpression of EDA in mice could aggravate the impaired glucose tolerance induced by HFD fed, while EDA knockdown could improve insulin sensitivity in diabetic mice. Further mechanism study found that the expression of EDA in mouse liver was regulated by peroxisome proliferator-activated receptor γ (PPARγ) and retinoid X receptor (RXR)-α, which further promoted c-Jun N-terminal kinase (JNK) activation and inhibited the serine phosphorylation of IRS1 in skeletal muscle, resulting in impaired insulin sensitivity of skeletal muscle in obesity. In addition, clinical studies have shown that the expression of EDA in liver was negatively correlated with glucose infusion rate (GIR). Finally, in another group of morbidly obese patients who received bariatric surgery intervention, the expression of EDA in liver was significantly decreased 1 year after surgery, while weight loss and insulin sensitivity were improved (14). Therefore, we speculate that EDA may be involved in the occurrence and development of diabetes through regulating insulin resistance, which needs further in-depth study to verify.

Previous studies have shown that hyperlipidemia was an important factor leading to insulin resistance (24, 25). Diabetic patients often have mixed dyslipidemia, which is mainly characterized by higher TG and lower HDL-c (25–27). In this study, compared with the normal control group, the T2DM group had higher TG and lower HDL-c levels. Furthermore, our results showed that the level of circulating EDA was positively correlated with TG and negatively correlated with HDL-C. However, our results were not completely consistent with a recent study by Yang et al. (13), which found that serum EDA levels in NAFLD patients were only correlated with HDL-c, but not with TG. This divergence may be due to the differences of sample size, drug, and diseases. In that study, 176 subjects were recruited, including 88 normal subjects and 88 patients with NAFLD while the subjects in our study were newly diagnosed T2DM patients. Although both are metabolic diseases and interact with each other, their pathogenesis and pathophysiology are different. In addition, none of the patients in our study received hypoglycemic and/or lipid-lowering drugs. We did not know whether patients with NAFLD in that study received medications or not. However, mounting evidence from recent fundamental studies suggested that EDA plays an important role in lipid metabolism. A clinical study demonstrated that the expression of liver EDA was positively correlated with liver fat content and visceral fat area and was positively correlated with histologically determined inflammation and steatosis score of nonalcoholic steatohepatitis (NASH) in 33 obese male patients (14). In animal studies, mice deficient EDA significantly reduced the increase of liver lipid droplets by HFD, decreased the content of hepatic TG, and reduced the levels of ALT and AST in HFD model mouse. What is more, in HepG2 cells, free fatty acids (FFA) intervention significantly increased the expression of EDA protein in cells and cell culture supernatant. Further mechanism studies showed that EDA gene knockout could increase the expression of carnitine palmitoyltransferase 1A (CPT1A), the key enzyme of fatty acid oxidation, and decrease the expression of sterol regulatory-element-binding protein-1c (SREBP-1c), acetyl coA carboxylase (ACC), and fatty acid synthase (FAS), thus reducing the accumulation of TG induced by FFA (13). Previous studies have shown that excessive fat accumulation can induce insulin resistance and destroy the function of islet cells (28), while insulin resistance can promote hyperlipidemia (4, 29), which is a vicious cycle. The latest research found that hepatokines play a very important role in it (7). Combined with the above results, high glucose and high fat can induce the secretion of EDA, and the increase of EDA further aggravates the disorder of glycolipid metabolism and insulin resistance. Therefore, we speculate that EDA, a new hepatokine, may also be involved in this vicious cycle.

Obesity, especially visceral obesity, not only affects body shape, but also is considered as a key risk factor for insulin resistance, diabetes, fatty liver, and even cardiovascular diseases (30, 31). In our study, we have shown that the serum level of EDA in the abdominal obesity group was significantly increased than that in the non-abdominal obesity group, and circulating EDA level was positively correlated with BMI and WHR, which is consistent with Yang et al. (13) in NFALD. The limitations of our research are also worth commenting on. First, this study was limited by a cross-sectional design and could not infer a causal relationship between elevated serum EDA levels and the development of T2DM. Second, all the study participants were recruited from a province, and the population was underrepresented. Third, our analysis was based on a single measurement in the blood, which may not reflect the EDA over time. Circulating EDA levels should be measured at different stages to further clarify its role in the pathogenesis of type 2 diabetes.

## Conclusions

Taken together, our results indicated for the first time that serum EDA levels were significantly increased in newly diagnosed T2DM patients. Moreover, circulating EDA concentrations were closely correlated with glycolipid metabolism and insulin resistance. Further studies are required to clarify the potential pathophysiological role of EDA in T2DM.

## Data Availability Statement

The original contributions presented in the study are included in the article/supplementary material. Further inquiries can be directed to the corresponding authors.

## Ethics Statement

The study was approved by the Biomedical Research Ethics Committee of Affiliated Hospital of Jiangsu University, Zhenjiang, China, and performed in accordance with the Declaration of Helsinki. The patients/participants provided their written informed consent to participate in this study.

## Author Contributions

GY and LY designed the study and drafted the manuscript. XD, ZC, and YL participated in the coordination of the whole work, analyzed data, and wrote the manuscript. LZ and HL interpreted the data, revised the manuscript, and recruited participants and collected data. XW, KC, CW, PZ, and ZZ participated in acquisition of data and analyzed the data. All authors contributed to the article and approved the submitted version.

## Funding

This research is funded by the National Natural Science Foundation of China (81870548, 82000809), the Social Development Project of Jiangsu Province (BE2018692), the Natural Science Foundation of Jiangsu Province (BK20191222), the Scientific Research Projects of Jiangsu Health and Family Planning Commission (Y2018109), the Science and Technology Planning Social Development Project of Zhenjiang City (SH2019041), and the ‘‘169-Phase V” Science Research Project of Zhenjiang.

## Conflict of Interest

The authors declare that the research was conducted in the absence of any commercial or financial relationships that could be construed as a potential conflict of interest.

## Publisher’s Note

All claims expressed in this article are solely those of the authors and do not necessarily represent those of their affiliated organizations, or those of the publisher, the editors and the reviewers. Any product that may be evaluated in this article, or claim that may be made by its manufacturer, is not guaranteed or endorsed by the publisher.
